# Identification of pregnancy-associated glycoproteins and alpha-fetoprotein in fallow deer (*Dama dama*) placenta

**DOI:** 10.1186/1751-0147-56-4

**Published:** 2014-01-13

**Authors:** Mathilde Bériot, Aline Flora Tchimbou, Olimpia Barbato, Jean-François Beckers, Noelita M de Sousa

**Affiliations:** 1 Laboratory of Animal Endocrinology and Reproduction, Faculty of Veterinary Medicine, University of Liege, B-4000 Liege, Belgium; 2 Department of Biopathological Veterinary Science, Faculty of Veterinary Medicine, University of Perugia, I-06126 Perugia, Italy

**Keywords:** Affinity chromatography, Fallow deer, N-terminal microsequencing, Pregnancy-associated glycoprotein, Vicia villosa agarose

## Abstract

**Background:**

This paper describes the isolation and characterization of pregnancy-associated glycoproteins (PAG) from fetal cotyledonary tissue (FCT) and maternal caruncular tissue (MCT) collected from fallow deer (*Dama dama*) pregnant females. Proteins issued from FCT and MCT were submitted to affinity chromatographies by using *Vicia villosa* agarose (VVA) or anti-bovine PAG-2 (R#438) coupled to Sepharose 4B gel. Finally, they were characterized by SDS-PAGE and N-terminal microsequencing.

**Results:**

Four distinct fallow deer PAG (fdPAG) sequences were identified and submitted to Swiss-Prot database. Comparison of fdPAG with PAG sequences identified in other ruminant species exhibited 64 to 83% identity. Additionally, alpha-fetoprotein was identified in fetal and maternal tissues.

**Conclusion:**

Our results demonstrate the efficacy of VVA and bovine PAG-2 affinity chromatographies for the isolation of PAG molecules expressed in deer placenta. This is the first report giving four specific amino acid sequences of PAG isolated from feto-maternal junction (FCT and MCT) in the Cervidae family.

## Background

The family Cervidae includes 40 species and constitutes the second most populous family of artiodactyls. There are four tribes: Cervinae, Muntiacinae, Hydropotinae, and Odocoileinae [1]. Fallow deer belongs to Cervinae tribe, and more specifically to *Dama* genus. The divergence between Cervidae and Bovidae was estimated to be 22.8 ± 4.7 MYA [2].

In north hemisphere countries, the breeding season of *Dama dama* occurs throughout October, but may be extended to November. The number of young is one but twins have been observed. The gestation period is 230–240 days; there is no evidence of delayed implantation [3]. The placentation is of oligocotyledonary type, with a maximum of 10 cotyledons in Cervidae family [4]. In histological point of view, placenta from deer is synepitheliochorial [5]. Binucleate cells are a constant characteristic of the trophoblast of the Cervidae. They carry a typical PAS-positive carbohydrate-protein complex [6,7]. They pass from the trophoblast into the crypt lining from the time the villus has occupied a crypt and represent 15-20% of the fetal trophectodermal cells [3].

Pregnancy-associated glycoproteins (PAG) also known as pregnancy-specific protein B (PSPB) or SBU3 antigen constitute a large family of placental glycoproteins [6,8,9]. They are members of the aspartic proteinase gene family and exhibit high sequence identities with other aspartic proteinases, such as pepsinogen, pepsin, chymosin, cathepsin D and E [10,11]. Based on their expression throughout the trophectoderm and on phylogenetic analyses, the PAG family members are separated into modern (PAG-I) and ancient groups (PAG-II) [12]. The modern PAG are expressed exclusively by binucleate cells whereas the ancient PAG are expressed by both mono- and binucleate trophoblastic cells [13]. The majority of PAG cDNA belongs to PAG-I group [13]. Divergence of PAG-I group is estimated to have taken place 52 ± 6 million years ago. Evolution of the PAG-II group is estimated to have occurred 87 ± 6 million years ago [12]. No precise function has been experimentally assigned yet for PAG molecules. However, according to different authors, the expression of PAG family members as early as Day 7 after fertilization suggests their potential role in cellular growth and differentiation, elongation, apposition, attachment, and placentogenesis processes [14]–[18].

PAG molecules were successfully purified and characterized in bovine (boPAG) [9], ovine (ovPAG) [19]–[21], caprine (caPAG) [22], water buffalo (wbPAG) [23,24], American bison (AmbPAG) [25] and European bison (EbPAG) [26] species. Huang *et al.*[27] were the first to report the partial characterization of PSPB molecules in moose (*Alces alces*) and elk (*Cervus canadensis*) placenta. However, as no N-terminal microsequences were reported in their study, it was not possible to compare sequences from Cervidae family with those obtained from common ruminant species. Still concerning Cervidae species, nine cDNA are known in white-tailed deer (*Odocoileus virginanus*) [28]. Besides, by using heterologous RIA systems, peripheral concentrations of PAG-PSPB molecules have been detected in the sera of moose, elk, mule deer (*Odocoileus hemionus*), white-tailed deer (*Odocoileus virginianus*), sika deer (*Cervus nippon*), caribou (*Rangifer tarandus*) and fallow deer (*Dama dama*) (revised by Sousa *et al.*[29]).

Depending on species, the degree of N-glycosylation of PAG varies from 10 to 17.83% [9,30]. Indeed, since the early 2000s, it was reported that lectins such as the agglutinins from *Vicia Villosa* agarose (VVA) bind to the N-acetyl galactosamine (GalNAc) of asparagine-linked glycans from PAG [31]. Therefore, VVA affinity chromatography has been applied with success to purify PAG molecules [23]–[25]. This paper describes the isolation and characterization of fallow deer PAG (fdPAG) proteins from placental extracts by using VVA and anti-PAG-2 affinity chromatographies.

## Materials and methods

### Collection of cotyledons

Uterus and placenta tissues were harvested from deer (n = 2) during the first half of gestation (110 days *post-coitum*). Females were taken for slaughter with the agreement of the local ethical authorities from University of Perugia. Immediately after the slaughter of females, uteri and placenta tissues were separated, extensively washed with 0.9% NaCl and frozen in liquid nitrogen.

### Measurement of total protein and PAG

Total protein concentrations (TP) of different fractions obtained during the isolation procedure were determined by Lowry method [32], with bovine serum albumine (BSA; ICN Biochemicals Inc., Aurora, OH, USA) as the standard.

Due to the absence of specific reagents for PAG measurements in Cervidae species, concentrations of immunoreactive fdPAG were monitored by two different heterologous radioimmunoassay (RIA) systems. Highly purified boPAG_67kDa_[9] was used as a tracer and standard in both RIA systems. Standard curves ranged from 0.8 to 100 ng/ml. Polyclonal antisera were raised in rabbits (R#) against purified bovine PAG-2 (anti-boPAG-2; R#438) [33]–[35] and caprine PAG (anti-caPAG_55+62kDa_; R#706) [22] antigens. Immunisation protocol was previously described by Vaitukaitis *et al.*[36].

For assay, each fraction was diluted in Tris–HCl buffer (0.025 M Tris, 0.01 M MgCl_2_, 0.01% (w/v) sodium azide, pH 7.5) containing 0.1% of BSA. Dilutions ranged from 1:1 until 1:100. Each dilution of the sample (0.1 ml) or standard (0.1 ml) was added to 0.2 ml of assay buffer. Samples were incubated overnight at 20-25°C with 0.1 ml of ^125^I-PAG (28,000 cpm) and 0.1 ml of each primary antibody (R#438 and R#706 used at initial dilutions of 1:4 000 and 1:120 000, respectively). The total volume of the reaction mixture was 0.5 ml. The next day, 1.0 ml of a double-antibody precipitation system was added to all the tubes except that for total count and a further 30 min incubation took place at room temperature (20-25°C). The end of the procedure was similar to that described previously by Barbato *et al.*[23].

### Isolation of placental proteins

#### Protein extraction

The whole procedure (homogenization, precipitation, centrifugation, and dialysis) was performed at 4°C with the exception of loading and elution of affinity chromatographies (realized at room temperature).

Fetal cotyledonary tissue (FCT; 173.5 g) and maternal caruncula tissue (MCT; 327.5 g) were minced separately. FCT and MCT were mixed five times (5 × 3 min) in potassium phosphate buffer (0.01 M KH_2_PO_4_ + 0.1 M KCl, pH 7.6) with a ratio 1:3 wt:vol (tissue:buffer). The pH was readjusted regularly to 7.6 with KOH. Phenylmethylsulphonylfluoride (PMSF, 0.2 mM), sodium azide (0.02% NaN_3_, wt:vol) and sodium EDTA (0.2% wt:vol) were added at the beginning of mixing. Each homogenate was stirred for 1 h and centrifuged at 20 000 × g during 50 min. The pellets (85.7 and 87.1 g for FCT and MCT, respectively) were taken to a second extraction. They were mixed (2 × 3 min) and homogenized (30 min) in 300 ml and 400 ml of potassium phosphate buffer, respectively. Additional PMSF (0.4 mM), sodium azide (0.04% wt:vol) and sodium EDTA (0.4% wt:vol) were added at the beginning of second extraction. The supernatants issued from the first extraction (0.62 and 1.2 l for FCT and MCT, respectively) were readjusted to pH 7.6 and let stand overnight. The next day, homogenates issued from second extraction were centrifuged (20 000 × g, 50 min). Supernatants from second extraction (0.34 and 0.42 l for FCT and MCT, respectively) were put together with those from first extraction. The pellets (66.7 and 80.4 g for FCT and MCT, respectively) were submitted to a third extraction in potassium phosphate buffer by using a glass tissue grinder. The two homogenates were centrifuged separately (20 000 × g, 50 min) and the supernatants (390 ml for both FCT and MCT) were added to those from previous extractions. The pellets were discarded.

#### Ammonium sulfate (A.S.) precipitation

The supernatants from the three extractions (FCT or MCT origins) were pooled. They were stirred and dry A.S. was slowly added to obtain 20% saturation solution (113 g/l) (0-20% A.S. fraction). After overnight precipitation, the homogenates were centrifuged at 20 000 × g, during 50 min. The pellets were eliminated. The supernatants (1.32 and 2.0 l for FCT and MCT, respectively) were stirred and dry A.S. was slowly added to obtain 40% saturation solution (121 g/l) (20-40% A.S. fraction). After 3 h precipitation, the homogenates were centrifuged at 20 000 × g, during 50 min and the pellets were discarded. The supernatants (1.34 and 2.1 l for FCT and MCT, respectively) were stirred and dry A.S. was slowly added to obtain 80% saturation solution (281 g/l) (40-80% A.S. fraction). After overnight precipitation, the homogenates were centrifuged at 20 000 × g, during 50 min. The supernatants (1.5 and 2.3 l for FCT and MCT, respectively) were discarded. The pellets (20 g for FCT and 37.2 g for MCT) were diluted in Tris–HCl buffer (0.01 M, pH 7.6) and dialyzed against the same buffer during 48 h. After dialyses, the solutions were centrifuged at 48 200 × g during 20 min. The pellets were eliminated and the supernatants (92 and 126 ml for FCT and MCT, respectively) were frozen.

#### Vicia villosa agarose affinity chromatography

The 40-80% A.S fraction from both FCT and MCT were submitted to VVA chromatography with the use of agarose-bound *Vicia villosa* lectin (Vector Laboratories, Burlingame, CA, USA). Fractions of 4 mL were collected. Optical density (OD) was measured at a wavelength of 280 nm.

Each chromatography was performed with 80 mg of placental protein previously dialyzed (16 h) in HEPES buffer (0.01 M, pH 7.6). The column (8 ml, 2.3 × 2 cm) was equilibrated with the same buffer. After loading, each sample (FCT or MCT) was gently mixed with VVA gel and then incubated overnight at room temperature (RT) into the VVA column. The unbound proteins were washed out with 80 ml of HEPES buffer (0.01 M, pH 7.6). Thereafter, HEPES buffer containing 0.15 M NaCl was loaded onto the column in order to eliminate weaker bound proteins. Proteins were eluted by using the same buffer (0.01 M HEPES + 0.15 M NaCl) added of 0.05 M GalNAc (AppliChem, Darmstadt, Germany). According to their OD, the fractions eluted in the same step (unbound or GalNAc-peak) were pooled, dialyzed (ammonium bicarbonate buffer 0.005 M, pH 8) and lyophilized. VVA gel was regenerated with NaCl (1 M, pH 3) between two consecutive chromatographies.

#### Antiserum 438 affinity chromatography

The 40-80% A.S fraction from both FCT and MCT were submitted to R#438 affinity chromatography. Firstly, total immunoglobulin fraction from the immunserum R#438 (Ig-438) were purified by ammonium sulphate precipitation and DEAE chromatography [37]. Briefly, 10 ml of crude R#438 were added of 2.5 g of dry A.S. The solution was let stand 20 h at RT. The next day, the solution was centrifuged (10 000 × g, 30 min) and the pellet was washed with 10 ml of 1.75 M A.S. solution. After an additional centrifugation, the pellet was solubilized with 15 ml of distilled water. Precipitated proteins were alternately dialyzed against four batches (5 l) of deionized water and ammonium acetate 0.05 M (pH 5.0). After the last dialysis, proteins were centrifuged (4 000 × *g*, 20 min) and the supernatant was loaded onto 2.5 ml of DEAE Sephadex A-50 column previously equilibrated with 0.05 M ammonium acetate (pH 5.0). Immunoglobulins were eluted in the non-adsorbed fraction by washing the column with 25 ml of ammonium acetate buffer. Eluted proteins (OD > 0.050) were pooled, dialyzed against 0.005 M ammonium bicarbonate buffer (pH 8) and lyophilized.

Sepharose 4B gel (Amersham Biosciences, Uppsala, Sweden) was activated with cyanogen bromide according to the technique previously described by Axen *et al.*[38]. Just prior coupled, Ig-438 (30 mg) were solubilized in 15 ml of NaHCO_3_ (0.1 M, pH 8.3) containing 0.5 M NaCl. Activated Sepharose 4B gel (10 ml) was added of Ig-438 (30 mg) and stirred 1 h at RT followed by 16 h at 4°C. The next day, unbound Ig-438 was washed out after centrifugation at 1 500 × *g* (20 min). Unbound sites were blocked by ethanolamine solution (1 M, pH 8). After standing 2 h at RT, the blocking solution was washed away by means of centrifugation. Finally unbound proteins were eliminated by six alternate washes with buffer A (0.1 M sodium acetate adjusted to pH 4 with acetic acid + 0.5 M NaCl) and buffer B (Tris–HCl 0.1 M adjusted to pH 8 + 0.5 M NaCl).

The Sepharose 4B Ig-438 column (0.7 × 5 cm, 2 ml) was equilibrated with PBS 0.05 M containing 0.15 M NaCl (pH 7.4). A total of 160 and 80 mg of proteins issued from either A.S 40-80% FTC or MCT fractions were loaded three consecutive times. Fractions of 1.5 ml were collected and protein content was monitored by measuring OD at 280 nm. The unbound proteins were eliminated after washing with 20 ml of PBS 0.05 M containing 0.15 M NaCl. In order to ensure the elimination of non-specific weakly bound proteins, a second wash was performed with 20 ml of PBS 0.05 M containing 0.3 M NaCl. Bound proteins were eluted by adding 0.1 M glycine solution adjusted to pH 2.8 with HCl. Before elution, 1.5 ml sodium bicarbonate buffer (0.1 M, pH 8.3) was added to each collection tube. According to their OD, the fractions belonging the same step (unbound or glycine eluted peak) were pooled together, dialyzed against ammonium bicarbonate buffer (0.005 M, pH 8) and lyophilized.

### Characterization of placental proteins

#### 1D-SDS PAGE

Fractions issued from different fractionation steps were denatured (5 min at 100°C) in Laemmli buffer containing 5% mercaptoethanol. Proteins were separated on a 12% polyacrylamide gel in the presence of SDS on a vertical slab gel system (0.15 × 8 × 7.3 cm). Electrophoresis was performed at 200 V during 40 min. Molecular weight standards (LMW Electrophoresis calibration Kit, Amersham Biosciences, Uppsala, Sweden) were run simultaneously. Proteins were visualized after Coomasie Brilliant Blue R250 staining (Merck, Darmstadt, Germany).

#### Western blot

Details on immunoblotting and Western blot techniques were previously described by Kiewisz *et al.*[25]. Briefly, proteins were transferred onto a nitrocellulose membrane (0.45 μm, Protran BA85; Schleicher and Schuell Biosciences, Dassel, Germany) after SDS-PAGE. The transfer was performed during 3 h at a constant voltage (60 V) on a TransBlot Cell Apparatus (BioRad, Hercules, CA, USA). Immediately after transfer, membranes were stained with Ponceau Red and extremities of bands corresponding to major proteins were pricked with a needle.

Immunoblotted proteins were probed with two distinct antisera raised against PAG: R#435 (anti-boPAG-2) or R#706 (anti-caPAG_55+62kDa_). Purification of boPAG-2 and caPAG_55+62kDa_ were described elsewhere [22,33]–[35]. The final dilution of each first antiserum was 1:100 (0.2 ml antiserum + 4.8 ml PAG-free serum + 15 ml Tris-buffer saline containing 1% BSA).

#### Transfer to PVDF membrane and N-terminal microsequence analysis

Proteins of interest (fractions eluted after VVA or R#438 affinity chromatographies) were separated after SDS-PAGE on a vertical slab gel system (0.1 × 16 × 14 cm; Protean Xi, BioRad). Gels were run at 15 mA/gel during migration in a stacking gel and at 27 mA/gel in the separating gel (12%). Molecular weight standards (LMW Electrophoresis calibration Kit, Amersham Bioscience, Uppsala, Sweden) were run simultaneously. Proteins were transferred onto 0.2 μm polyvinylidene difluoride (PVDF) membranes (BioRad) for N-terminal microsequencing. The transfer was carried out on a Transblot Cell Apparatus (BioRad) at a constant voltage (60 V) during 2 h 30 min. The PVDF membrane was stained with 0.2% (w/v) Coomassie Brilliant Blue R-250 for 5 min and destained 3 times in 50% methanol solution. After PVDF membranes were dried, proteins were excised and subjected to Edman degradation on a pulsed liquid-phase protein sequencer (Procise 492 Applied Byosystems, Foster City, CA, USA).

The N-terminal sequences obtained in fallow deer have been deposited in the EMBL-EBI database (Swiss-Prot: C0HJC7, C0HJC8, C0HJC9, C0HJD0). N-terminal sequences were compared to those described in databank in order to detect homologies with other isolated native proteins or those deduced from cDNA (Blast, NCBI).

## Results

### Isolation of fdPAG from FCT and MCT

After extraction, concentrations of PAG measured by RIA remained very low in both FCT and MCT (data not shown). They were proportionally higher after 3rd extraction than 1st + 2nd extractions.

Concerning ammonium sulphate precipitations, as shown in Table 1, in both FCT and MCT tissues, the ratio of equivalent fdPAG measured by using RIA-438 to TP reached higher values after precipitation at 40-80% A.S. saturation. However, when using RIA-706, the ratio of equivalent fdPAG to TP remained very low (<0.06%) in both tissues.

**Table 1:** Total protein (TP) and equivalent fallow deer PAG (fdPAG) detected by two heterologous RIA systems

	**Fetal cotyledonary tissue**			**Maternal caruncular tissue**		
		**fdPAG (mg) and [PAG/TP ratio (%)]**			**fdPAG (mg) and [PAG/TP ratio (%)]**	
Purification step	TP (mg)	RIA-438	RIA-706	TP (mg)	RIA-438	RIA-706
20-40% A.S.	1,401.6	3.16 [0.23]	0.12 [0.01]	1,956.5	1.23 [0.06]	0.06 [0]
40-80% A.S.	2,014.9	10.59 [0.53]	0.44 [0.02]	815.2	3.95 [0.48]	0.31 [0.04]

*RIA-438*, Use of anti-boPAG-2; *RIA-706*, Anti-caprine PAG_55+62kDa_.

**Figure 1:**
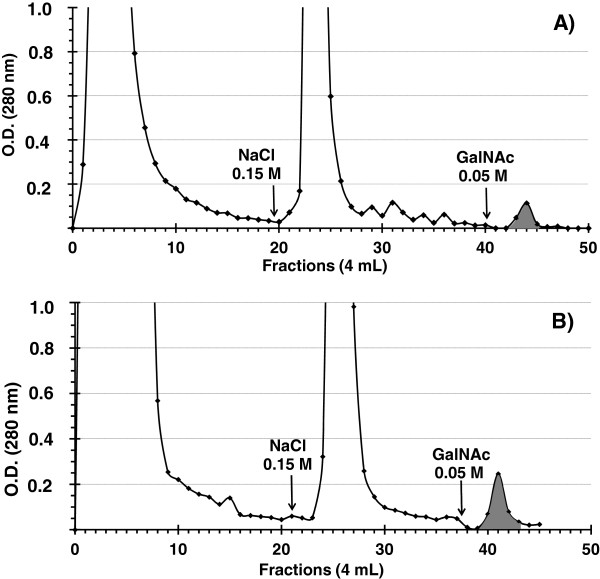
***Vicia villosa *chromatographic profiles from fallow deer.** Fractions were issued from fetal cotyledonary **(A)** and maternal caruncula **(B)** tissues. The column (2.3 × 2 cm; 8 ml) was previously equilibrated with 0.01 M HEPES buffer (pH 7.6). The elution with 0.15 M NaCl or 0.05 M GalNAc buffer (containing 0.15 M NaCl) were designated by arrow. The pooled fractions are in gray.

The elution profiles of VVA affinity chromatography are shown in Figure 1A and 1B. After addition of 0.05 M GalNAc, glycoproteins from FCT and MCT were eluted in one major peak.

**Figure 2:**
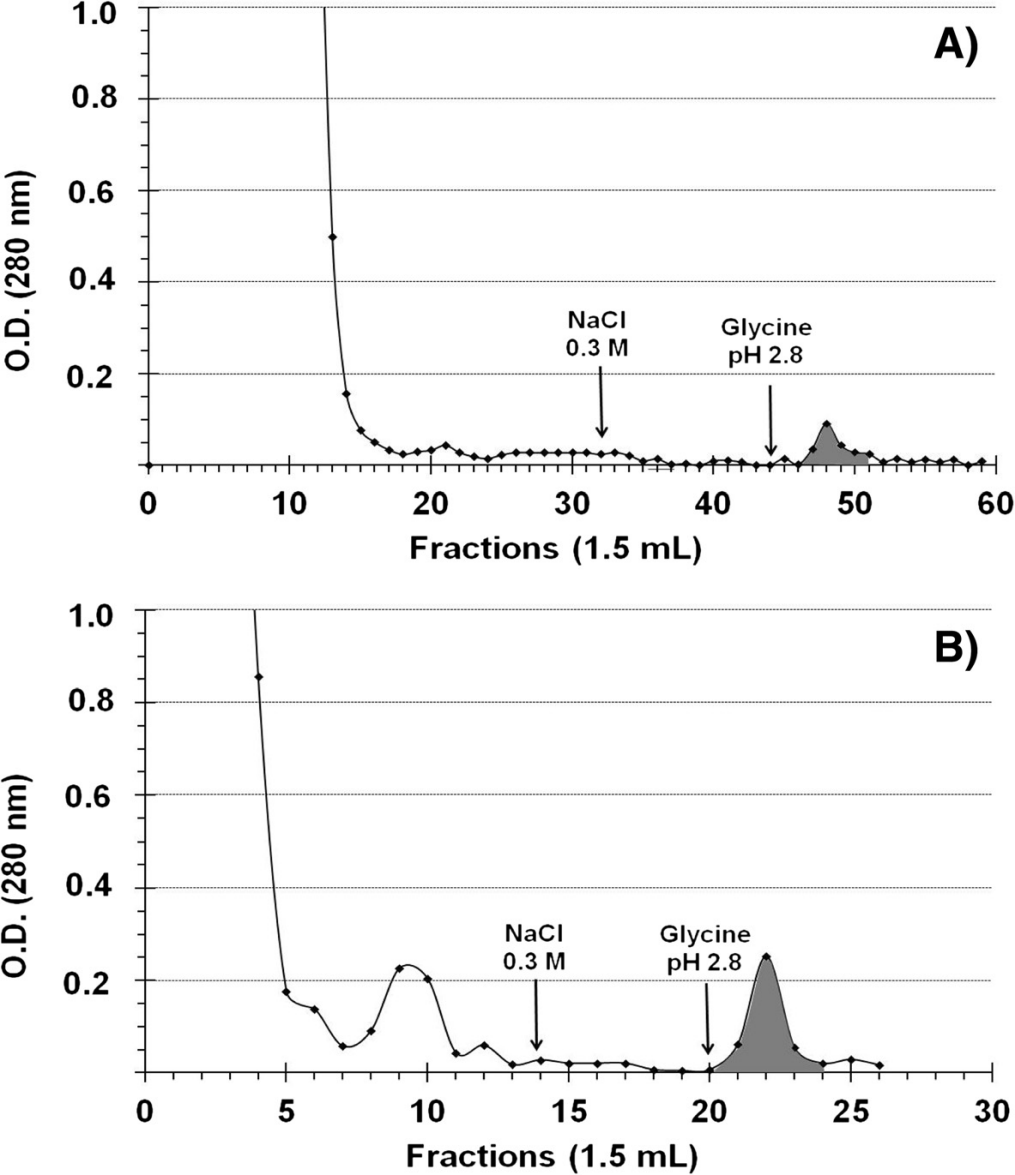
**Ig-438 affinity chromatography profiles from fallow deer.** Fractions were issued from fetal cotyledonary **(A)** and maternal caruncula **(B)** tissues. Column (0.7 × 5 cm, 2 ml) was previously equilibrated with PBS 0.05 M containing 0.15 M NaCl (pH 7.4). Unbound proteins were eliminated after washing with PBS 0.05 M containing 0.15 M NaCl, followed by washing with PBS 0.05 M containing 0.3 M NaCl. Bound proteins were eluted by adding 0.1 M glycine solution adjusted to pH 2.8 with HCl. Before elution, 1.5 ml sodium bicarbonate buffer (0.1 M, pH 8.3) was added to each collection tube.

Finally, with regard to Sepharose 4B Ig-438 affinity chromatography, no protein was eluted after washing with PBS containing 0.3 M NaCl. Addition of glycine-HCl solution (pH 2.8) allowed proteins eluting in one major peak (Figure 2A and 2B).

### Characterization of fdPAG from FCT and MCT

**Figure 3:**
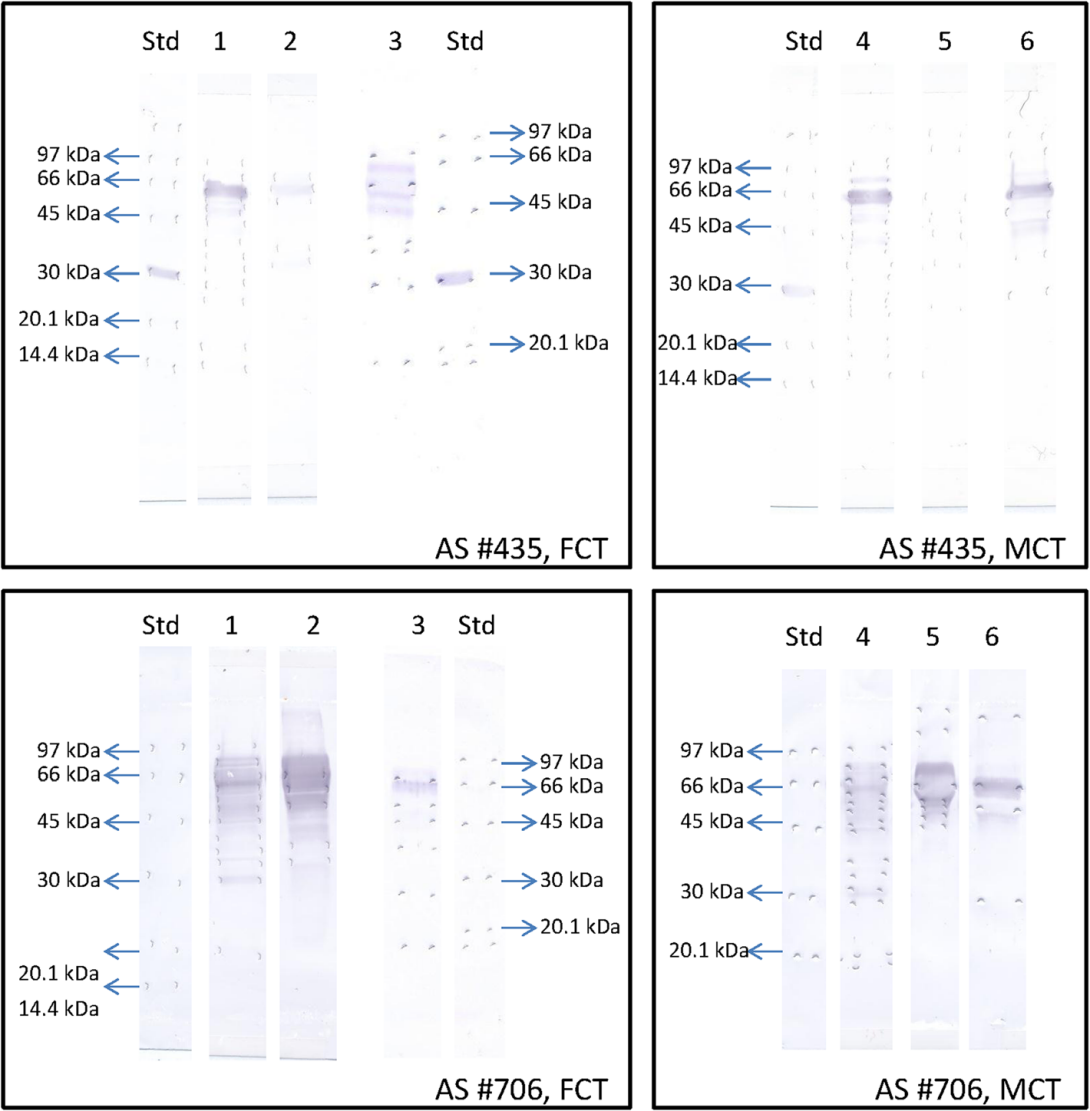
**Western blot of fdPAG proteins extracted from fetal cotyledonary (FCT) and maternal caruncular (MCT) tissues.** Two polyclonal antisera raised against boPAG-2 (AS#435) or caprine PAG_55+62kDa_ (AS#706) were used as primary antiserum at 1:100 dilution. Molecular weight standards (kDa; 7 μg/lane) were loaded on the right or left position of figures. Lane 1: A.S. 40-80% fraction from FCT; Lane 2: VVA eluted peak from FCT; Lane 3: eluted peak from Sepharose 4B Ig-438 affinity chromatography from FCT; Lane 4: A.S. 40-80% from MCT; Lane 5: VVA peak from MCT; Lane 6: eluted peak from Sepharose 4B Ig-438 affinity chromatography from MCT. Fifty μg were loaded in lanes 1 and 4; 30 μg were loaded on lanes 2, 3, 6 and 7.

As shown in Figure 3, after precipitation at 40-80% A.S. of FCT, a single immunoreactive protein (60 kDa) could be observed when using R#435 for Western blot. By using the same antiserum in MCT, two major immunoreactives bands (63 and 66 kDa) were observed after precipitation at 40-80% A.S. saturation. Immunoreactive proteins were also observed in the eluted peak issued from Sepharose 4B Ig-438 affinity chromatography of MCT.

**Figure 4:**
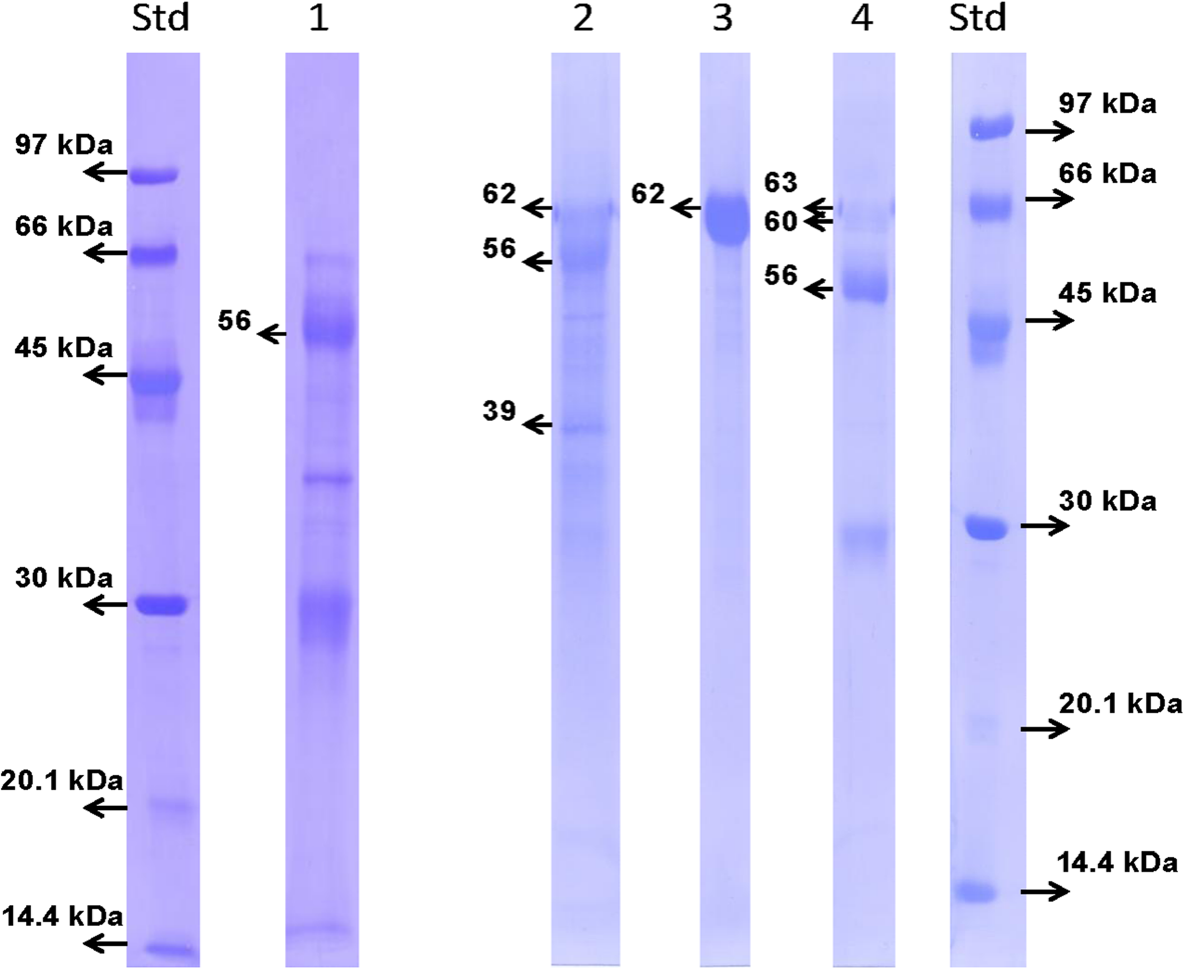
**Coomassie blue stained PVDF membrane after SDS-PAGE.** Low molecular weight standards (kDa; 7 μg/lane) were loaded on the right and the left side of the figure. Lane 1: eluted peak from Sepharose 4B Ig-438 affinity chromatography from FCT; Lane 2: VVA eluted peak from FCT; Lane 3: eluted peak from Sepharose 4B Ig-438 affinity chromatography from MCT; Lane 4: VVA peak from MCT. Fifty to 75 μg were loaded in each lane.

When using R#706, multiple immunoreactive bands (MM ranging from 45 to 70 kDa) could be observed as well after precipitation at 40-80% A.S. saturation than in eluted peaks from VVA and Sepharose 4B Ig-438 affinity chromatographies. Molecular masses were slightly lower after Western blot than after immunobloting on PVDF membrane and Coomassie staining (58 to 63 kDa) (Figure 4).

Several proteins issued from affinity chromatography and immunoreactive after Western blot with R#435 or R#706 were submitted to N-terminal amino acid sequencing (Figure 4). As described in Table 2, four N-terminal sequences exhibited quite high amino acid identities with PAG molecules identified in other ruminant species. These proteins were named according to their molecular masses: fdPAG_62kDa__A, fdPAG_56kDa__B, fdPAG_39kDa__C and fdPAG_56kDa__D. Moreover, three proteins exhibited high sequence identity with alpha-fetoprotein and another one with albumin.

**Table 2:** **Molecular masses and N-terminal sequences of proteins isolated from fallow deer ( *Dama dama *) placenta**

**Origin of tissue**	**Affinity chromatography used for isolation**	**MM (kDa)**	Sequenced fragment*	**Protein**	**Accession number**
FCT	VVA	62	YQKSSPGSNITT	fdPAG_62kDa__A	C0HJC7
		56	YQTSSPGSNITIGPL	fdPAG_56kDa__B	C0HJC8
		39	DVGPSTLANN	fdPAG_39kDa__C	C0HJC9
	Antiserum R#438 Sepharose	56	IPLDTIAGYKE	Alpha-fetoprotein	C0HJD1
MCT	VVA	62	DTHKSEIAHR	Albumin	C0HJD2
	Antiserum R#438 Sepharose	63	IPLDTIAGY	Alpha-fetoprotein	C0HJD1
		60	IPLDTIAGYKE	Alpha-fetoprotein	C0HJD1
		56	SLRKMHALGET	fdPAG_56kDa__D	C0HJD0

*FCT*, Fetal cotyledonary tissue; *MCT*, Maternal caruncular tissue; *MM*, Molecular mass.

* Amino acid microsequence analysis was performed by the automated Edman degradation method.

Sequence comparison of fdPAG (Table 2) with those previously identified in ruminant species ranged from 64 to 83% (Table 3). One PAG sequence exhibited a lower molecular mass (fdPAG_39kDa__C) and exhibited high amino acid identity with internal fragments of boPAG-6, boPAG-4 and ovPAG-1. The three other sequences appear clearly to correspond to N-terminal PAG termini (fdPAG_62kDa__A, fdPAG_56kDa__B and fdPAG_56kDa__D).

**Table 3:** Comparison of N-terminal amino acid sequences isolated from fallow deer with those inferred from DNA databases

**Protein**	**Accession number**	**1**	**2**	**3**	**4**	**5**	**6**	**7**	**8**	**9**	**10**	**11**	**12**	**13**	**14**	**15**	**Amino acid identity (Id)**
fdPAG_62kDa__A	C0HJC7	Y	Q	K	S	S	P	G	S	N	I	T	T				
DeerPAG-1	Q6R6P2	Y	**K**	**T**	S	S	P	G	S	N	I	T	T				10/12 (83%)
DeerPAG-2	Q6R6P1	Y	**K**	**T**	S	S	P	G	S	N	I	**A**	**A**				8/12 (67%)
fdPAG_56kDa__B	C0HJC8	Y	Q	T	S	S	P	G	S	N	I	T	I	G	P	L	
DeerPAG-1	Q6R6P2	Y	**K**	T	S	S	P	G	S	N	I	T	**T**	**H**	P	L	12/15 (80%)
Predict ovPAG-1 like	XP_004019614.1	**S**	Q	**I**	S	S	**R**	G	S	N	I	T	I	**H**	P	L	11/15 (73%)
boPAG-5	O46493	**S**	Q	**I**	S	S	**R**	G	S	N	I	T	I	**H**	P	L	11/15 (73%)
buPAG-10	E3UMT6	**S**	Q	**I**	S	S	**R**	G	S	N	I	T	I	**H**	P	L	11/15 (73%)
DeerPAG-2	Q6R6P1	Y	**K**	T	S	S	P	G	S	N	I	**A**	**A**	**Y**	P	L	11/15 (73%)
boPAG-17	Q9TTV7	**S**	Q	**I**	S	S	**R**	G	S	N	**L**	T	I	**H**	P	L	10/15 (67%)
fdPAG_39kDa__C	C0HJC9	D	V	G	P	S	T	L	A	N	N						
boPAG-6	O46494	**I**	V	G	P	S	T	L	**V**	N	N						8/10 (80%)
boPAG-4	O46492	**I**	V	G	P	S	T	L	**V**	N	N						8/10 (80%)
ovPAG-1	XP_004019891.1	**I**	**Q**	G	P	S	T	L	**V**	N	N						7/10 (70%)
fdPAG_56kDa__D	C0HJD0	S	L	R	K	M	H	A	L	G	E	T					
boPAG-12	O46500	**P**	L	R	K	M	**K**	**T**	L	**R**	E	T					7/11 (64%)
Alpha-fetoprotein	C0HJD1	I	P	L	D	T	I	A	G	Y	K	E					
Ovine alpha-fetoprotein	NP_001009802.1	I	P	L	D	**P**	I	A	G	Y	K	E					10/11 (91%)
Bovine alpha-fetoprotein	NP_776409.1	I	P	L	D	**P**	**V**	A	G	Y	K	E					9/11 (82%)
Yak alpha-fetoprotein	ELR45247.1	I	P	L	D	**P**	**V**	A	G	Y	K	E					9/11 (82%)
Albumin	C0HJD2	D	T	H	K	S	E	I	A	H	R						
Bovine albumin	AAA51411.1	D	T	H	K	S	E	I	A	H	R						10/10 (100%)
Caprine albumin	P85295	D	T	H	K	S	E	I	A	H	R						10/10 (100%)

*fd*, Fallow deer; *Deer*, White-tailed deer; *bo*, Bovine; *ov*, Ovine; *bu*, Water buffalo.

N-terminal amino acid sequences were submitted to Swiss-Prot data bank. Fallow deer pregnancy-associated glycoproteins (fdPAG), alpha-fetoprotein and albumin were aligned with purified proteins or polypeptide precursors deduced from DNA databases. Boldface letters indicate divergence in amino acid regarding the fallow deer protein.

## Discussion

This paper describes the first isolation and N-terminal microsequencing of PAG molecules from fallow deer. In order to avoid time-related degradation of PAG, our purification protocol was simplified in three main steps: extraction, ammonium sulfate precipitation and affinity chromatographies (VVA or Sepharose 4B Ig-438). As described by Huang *et al.*[27], thawing and freezing moose and elk cotyledons three times and stirring with sand to abrade the surface of binucleate cells was a helpful procedure for PSPB purification. In the present work, the use of tissue grinder during the third extraction of fallow deer tissues was helpful for the recovery of placental proteins from FCT and MCT. Together, these findings suggest that in Cervidae species, PAG-PSPB molecules could be strongly related to fetal and maternal membranes.

Despite PAG molecules are synthesized in the outer epithelial cell layer (chorion) of fetal cotyledons, purification was performed in both FCT and MCT. Indeed, by using immonocytochemical techniques, PAG-immunoreactivity has been demonstrated not only in fetal cotyledonary but also in maternal caruncular connective tissues in bovine species [14]. Moreover, amount of immunoreactive PAG measured by RIA in *Cervus elaphus* (red deer) extracts was higher in MCT than in FCT [39]. These findings can result from the migration of fetal binucleate cells toward the maternal junction.

Low PAG concentrations were observed in both FCT and MCT extracts (use of heterologous RIA-438 and RIA-706). In the same way, concentrations of PAG measured in plasma from pregnant fallow deer are very low [40]–[42]. Low concentrations can be related to the use of a bovine PAG-PSPB preparation as standard and tracer. According to Brandt *et al.*[28], PAG from white-tailed deer are mostly from PAG-II group. This observation was confirmed by results presented in Table 1, which reports that immunoreactivity of fdPAG is much higher when measured by using the anti-boPAG-2 antiserum (R#438).

Molecular masses of three fdPAG (calculated after Coomassie staining) ranged from 56 to 62 kDa. They are in the range of those described in other species [43] and are slightly lower than those observed after Western blot. In previous works, the same phenomenon was observed in both buffalo [23,24] and American bison [25]. Unfortunately, the explanation of such a difference is not known. However, it can be hypothesized that the calculation of molecular mass after Western blot is less precise due to the greater width of bands. Accordingly, we choose to assign proteins on the basis of their molecular mass calculated after Coomassie blue staining.

Interestingly, N-terminal extremities of fdPAG_62kDa__A and fdPAG_56kDa__B did not show the highly conserved RGS- amino acid residues previously reported for other ruminant species [19]–[26]. However, they fit with the PGS- sequence described for Deer PAG-1 and -2 in white-tailed deer [28]. In their sequenced part, identities of proteins isolated in fallow deer and identified in white-tailed deer ranged from 67 to 80%. This discrepancy can be partially explained by the phylogenetic divergence between *Dama dama* and *Odocoileus virginianus*, estimated to be 7.17 MYA [44].

Concerning belonging of fallow deer PAG to modern (PAG-I) or ancient (PAG-II) groups, as described in Table 3, N-termini of fdPAG_62kDa__A and fdPAG_56kDa__B exhibit homologies higher than 70% with different proteins from PAG-I group previously identified in white-tailed deer [28], cow [9,10], and ewe [10] placentas. On the other side, fdPAG_56kDa__D N-terminal sequence was identified as relatively close to boPAG-12 (PAG-II group). Nevertheless, as N-terminal micro-sequencing only refers to a limited number of residues, we could not perform the comparison of the whole sequences from *Dama dama* with PAG molecules deduced from cDNA and described in other ruminant and porcine species [43].

An additional protein (fdPAG_39kDa__C) issued from VVA chromatography (FCT) exhibited a lower molecular mass. This protein showed a high amino acid identity with internal fragments of boPAG-6, boPAG-4 and ovPAG-1, indicating that it corresponds to an internal fragment of PAG. A similar finding was described by Doré *et al.*[45] concerning the porcine basic protein, which corresponded to an internal fragment of porcine PAG.

Huang *et al.*[27] described the use of an affinity chromatography developed by antiserum anti-bovine PSPB for isolation of PSPB molecules from moose and elk placenta. However, they did not characterize the proteins they could obtain. In the present study, we performed affinity chromatographies on FCT and MCT by using an antiserum raised against a boPAG-2 preparation [33-35]. Western blot analysis highlighted immunoreactivity of several proteins issued from Sepharose 4B Ig-438 affinity chromatography with both R#435 and R#706. This chromatography allowed the successful identification of one fdPAG while three other sequenced proteins corresponded to alpha-fetoprotein (AFP, also known as fetuin A or alpha-2HS glycoprotein). AFP is the major serum protein in fetal ruminants, pigs, as well in humans and rodents [46]. This protein was firstly described in 1944 by Pedersen [47] and further characterized by Spiro [48]. AFP is synthesized and secreted by the fetal liver [49] and to a lesser extent the placenta, kidneys and the tongue [50]. Molecular mass of AFP is very similar to those from PAG-PSPB molecules. It ranges from 51 to 67 kDa depending on carbohydrate content (6 to 8%). However, in contrast to PAG-PSPB, the concentrations of AFP do not increase in maternal circulation during gestation [49] and cannot be used for pregnancy diagnosis in cattle [51,52].

It is noteworthy that Butler *et al.*[8] co-purified AFP and PAG-PSPB from bovine placental tissues collected between Days 16 and 280. Thus, it cannot be excluded that boPAG-2 antigen (used to generate R#438) could be contaminated by AFP. We hypothesize that contamination of boPAG-2 preparation with AFP may explain at least partially the poor ability of Sepharose 4B Ig-438 affinity chromatography to isolate PAG in fallow deer species. However, other factors such as phylogenetic divergence between PAG molecules isolated in fallow deer and bovine species cannot be excluded.

Regarding VVA affinity chromatography, the major protein obtained from MCT corresponded to the N-terminal sequence of serum albumin. Both albumin and AFP belong to albuminoidal gene superfamily. They are known to bind and/or transport a multitude of ligands, such as bilirubin, fatty acids, steroids, heavy metals and others [53]. Mature bovine serum albumin (BSA) is a single chain non-glycosilated polypeptide (583 amino acids long; accession number P02769) and contains three structural domains. BSA displays a molecular mass of 66 kDa. In fallow deer, apparent molecular mass was slightly lower after SDS-PAGE (62 kDa). As serum albumin is the most abundant protein of the circulation [54], it is hypothesized that fallow deer albumin detected in extracts of extraembryonic membranes might have been due to its higher abundance in maternal blood within the cotyledons.

When data of Table 3 are considered, it appears that albumin shows the same N-terminal sequence in cow, goat and fallow deer, whereas alpha-fetoprotein and PAG exhibits lower identities with other ruminants (82 to 91% and 64 to 83%, respectively). This observation can be related to the rapid evolution of PAG having lead to the high diversity observed in ruminant species [11,12,55].

Concerning biological aspects of PAG, an interesting review by Roberts *et al.*[55] suggested that PAG could sequester or transport small peptides in their binding cleft (6–8 amino acids long) [11]. An alternative hypothesis was recently described by Telugu *et al.*[56] who reported that placental aspartic proteinases might participate in placental remodeling by means of proteolytic digestion of endocytosed proteins in the uterine lumen. In the present study, N-terminal sequences of PAG extracted from *Dama dama* exhibited high identities with those from both modern (PAG-I, incapable of acting as proteolytic enzymes) and ancient groups (PAG-II, predicted to possess proteolytic activity). Moreover, our investigation confirmed that deer PAG molecules require abrasive extraction from cotyledonary tissue. Together, these findings claim for PAG as participating to a complex network of tight-junction-associated proteins communicating at feto-maternal interface.

## Conclusion

This is the first study describing the isolation and characterization of PAG from fallow deer placenta. The use of VVA and Sepharose 4B Ig-438 affinity chromatographies allowed the identification of four PAG molecules. Analysis of N-terminal sequences revealed high sequence identity with PAG from other ruminant species.

## Competing interests

The authors declare that they have no competing interests.

## Authors’ contributions

MB performed experimental work, data analysis and drafted the manuscript. AFT assisted in the design of study and participated in carrying out chromatography and radioimmunoassay. OB participated in the design of the study. JFB conceived the design of the study, coordinated the work and helped in writing the manuscript. NMS participated in carrying out PAG purification and sequence analysis, and had important input into and participation in writing the manuscript. All authors read and approved the final version of the manuscript.

## References

[B1] Gilbert C Ropiquet A Hassanin A Mitochondrial and nuclear phylogenies of Cervidae (Mammalia, Ruminantia): Systematics, morphology, and biogeography Mol Phylogen Evol 2006 56 101 117 10.1016/j.ympev.2006.02.017 16584894

[B2] Kumar S Hedges B A molecular timescale for vertebrate evolution Nature 1998 56 917 920 10.1038/31927 9582070

[B3] Hamilton WJ Harrison RJ Young BA Aspects of placentation in certain Cervidae J Anat 1960 56 1 33 14399289 PMC1244412

[B4] Sinha AA Seal US Erickson AW Mossman HW Morphogenesis of the fetal membranes of the white-tailed deer Am J Anat 1969 56 201 242 10.1002/aja.1001260206 5362274

[B5] Lee CS Wooding FB Morgan G Quantitative analysis of intraepithelial large granular lymphocyte distribution and maternofetal cellular interactions in the synepitheliochorial placenta of the deer J Anat 1995 56 Pt 2 445 460 7592007 PMC1167439

[B6] Lee CS Gogolin-Ewens K Brandon MR Comparative studies on the distribution of binucleate cells in the placentae of the deer and cow using the monoclonal antibody, SBU-3 J Anat 1986 56 163 179 3693070 PMC1261555

[B7] Wooding FB Morgan G Adam CL Structure and function in the ruminant synepitheliochorial placenta: central role of the trophoblast binucleate cell in deer Microsc Res Tech 1997 56 88 99 10.1002/(SICI)1097-0029(19970701/15)38:1/2<88::AID-JEMT10>3.0.CO;2-A 9260840

[B8] Butler JE Hamilton WC Sasser RG Ruder CA Hass GM Williams RJ Detection and partial characterization of two bovine pregnancy-specific protein Biol Reprod 1982 56 925 933 10.1095/biolreprod26.5.925 6807365

[B9] Zoli AP Beckers JF Wouters-Ballman P Closset J Falmagne P Ectors F Purification and characterization of a bovine pregnancy-associated glycoprotein Biol Reprod 1991 56 1 10 10.1095/biolreprod45.1.1 1908709

[B10] Xie SC Low BG Nagel RJ Kramer KK Anthony RV Zoli AP Beckers JF Roberts RM Identification of the major pregnancy-specific antigens of cattle and sheep as inactive members of the aspartic proteinase family Proc Natl Acad Sci U S A 1991 56 10247 10251 10.1073/pnas.88.22.10247 1946444 PMC52905

[B11] Guruprasad K Blundell TL Xie S Green J Szafranska B Nagel RJ McDowell K Baker CB Roberts RM Comparative modeling and analysis of amino acid substitutions suggests that the family of pregnancy-associated glycoproteins includes both active and inactive aspartic proteinases Prot Eng 1996 56 849 856 10.1093/protein/9.10.849 8931124

[B12] Hughes AL Green JA Garbayo JM Roberts RM Adaptive diversification within a large family of recently duplicated, placentally expressed genes Proc Natl Acad Sci U S A 2000 56 3319 3323 10.1073/pnas.97.7.3319 10725351 PMC16237

[B13] Green J Xie S Quan X Bao B Gan X Mathialagan N Roberts RM Pregnancy-associated bovine and ovine glycoproteins exhibit spatially and temporally distinct expression patterns during pregnancy Biol Reprod 2000 56 1624 1631 10.1095/biolreprod62.6.1624 10819764

[B14] Wooding FB Roberts RM Green JA Light and electron microscope immunocytochemical studies of the distribution of pregnancy-associated glycoproteins (PAGs) throughout pregnancy in the cow: possible functional implications Placenta 2005 56 807 827 10.1016/j.placenta.2004.10.014 16226131

[B15] Hashizume K Analysis of uteroplacental-specific molecules and their functions during implantation and placentation in the bovine J Reprod Dev 2007 56 1 11 10.1262/jrd.18123 17332695

[B16] Mamo S Mehta JP McGettigan P Fair T Spencer TE Bazer FW Lonergan P RNA sequencing reveals novel gene clusters in bovine conceptuses associated with maternal recognition of pregnancy and implantation Biol Reprod 2011 56 1143 1151 10.1095/biolreprod.111.092643 21795669

[B17] Thompson IM Cerri RL Kim IH Ealy AD Hansen PJ Staples CR Thatcher WW Effects of lactation and pregnancy on metabolic and hormonal responses and expression of selected conceptus and endometrial genes of Holstein dairy cattle J Dairy Sci 2012 56 5645 5656 10.3168/jds.2011-5113 22863093

[B18] Hue I Degrelle SA Turenne N Conceptus elongation in cattle: Genes, models and questions Anim Reprod Sci 2012 56 19 28 10.1016/j.anireprosci.2012.08.007 22921267

[B19] Xie S Green J Bao B Beckers JF Valdez KE Hakami L Roberts RM Multiple pregnancy-associated glycoproteins are secreted by day 100 ovine placental tissue Biol Reprod 1997 56 1384 1393 10.1095/biolreprod57.6.1384 9408244

[B20] El Amiri B Remy B Sousa NM Joris B Otthiers NG Perenyi Z Banga Mboko H Beckers JF Isolation and partial characterization of three pregnancy-associated glycoproteins from ewe placenta Mol Reprod Dev 2003 56 199 206 10.1002/mrd.10246 12506352

[B21] El Amiri B Remy B de Sousa NM Beckers JF Isolation and characterization of eight pregnancy-associated glycoproteins present at high levels in the ovine placenta between day 60 and day 100 of gestation Reprod Nutr Dev 2004 56 169 181 10.1051/rnd:2004025 15460157

[B22] Garbayo JM Remy B Alabart JL Folch J Wattiez R Falmagne P Beckers JF Isolation and partial characterization of a pregnancy-associated glycoprotein family from the goat placenta Biol Reprod 1998 56 109 115 10.1095/biolreprod58.1.109 9472930

[B23] Barbato O Sousa NM Klisch K Clerget E Debenedetti A Barile VL Malfatti A Beckers JF Isolation of new pregnancy-associated glycoproteins from water buffalo (*Bubalus bubalis*) placenta by *Vicia villosa* affinity chromatography Res Vet Sci 2008 56 457 466 10.1016/j.rvsc.2008.01.004 18308351

[B24] Barbato O Melo de Sousa N Barile VL Canali C Beckers JF Purification of pregnancy-associated glycoproteins from late-pregnancy Bubalus bubalis placentas and development of a radioimmunoassay for pregnancy diagnosis in water buffalo females BMC Vet Res 2013 56 89 10.1186/1746-6148-9-89 23634647 PMC3661400

[B25] Kiewisz J Melo de Sousa N Beckers JF Vervaecke H Panasiewicz G Szafranska B Isolation of pregnancy-associated glycoproteins from placenta of the American bison (*Bison bison*) at first half of pregnancy Gen Comp Endocrinol 2008 56 164 175 10.1016/j.ygcen.2007.04.011 17543308

[B26] Kiewisz J Melo de Sousa N Beckers JF Panasiewicz G Gizejewski Z Szafranska B Identification of multiple pregnancy-associated glycoproteins (PAGs) purified from the European bison (Eb; *Bison bonasus* L.) placentas Anim Reprod Sci 2009 56 229 250 10.1016/j.anireprosci.2008.04.021 18538513

[B27] Huang F Cockrell DC Stephenson TR Noyes JH Sasser RG Isolation, purification, and characterization of pregnancy-specific protein B from elk and moose placenta Biol Reprod 1999 56 1056 1061 10.1095/biolreprod61.4.1056 10491644

[B28] Brandt GA Parks TE Killian G Ealy AD Green JA A cloning and expression analysis of pregnancy-associated glycoproteins expressed in trophoblasts of the white-tail deer placenta Mol Reprod Dev 2007 56 1355 1362 10.1002/mrd.20669 17393426

[B29] Sousa NM Figueiredo JR Beckers JF Renaville R, Burny A Placental proteins in ruminants: biochemical, physiological and zootechnical aspects Biotechnology in Animal Husbandry 2001 The Netherlands: Kluwer Academic Publishers 179 208

[B30] Atkinson YH Gogolin-Ewens KJ Hounsel EF Davies MJ Brandon MR Seamark RF Characterization of placentation-specific binucleate cell glycoproteins possessing a novel carbohydrate J Biol Chem 1993 56 26679 26685 8253801

[B31] Klisch K de Sousa NM Beckers JF Leiser R Pich A Pregnancy-associated glycoprotein-1,- 6, -7, and -17 are major products of bovine binucleate trophoblast giant cells at midpregnancy Mol Reprod Dev 2005 56 453 460 10.1002/mrd.20296 15822115

[B32] Lowry OH Rosenbrough NJ Farr AL Randall RJ Protein measurement with the Folin phenol reagent J Biol Chem 1951 56 265 275 14907713

[B33] Beckers JF Dewulf M Verstegen J Wouters-Ballman P Ectors F Isolation of a bovine chorionic gonadotrophin (bCG) Theriogenology 1988 56 s218 10.1016/0093-691X(88)90046-5

[B34] Xie S Low BG Nagel RJ Beckers JF Roberts RM A novel glycoprotein of the aspartic proteinase gene family expressed in bovine placental trophectoderm Biol Reprod 1994 56 1145 1153 10.1095/biolreprod51.6.1145 7534122

[B35] Beckers JF Roberts RM Zoli AP Ectors F Derivaux J Molecules of the family of aspartic proteinases in the placenta of ruminants: hormones or proteins? Bull Mem Acad Royale de Med Belg 1994 56 355 367 7550037

[B36] Vaitukaitis J Robbins JB Nieschlag E Ross GT A method for producing specific antisera with small doses of immunogen J Clin Endocrinol Metab 1971 56 988 991 10.1210/jcem-33-6-988 5316354

[B37] Harboe N Ingild A Immunization, isolation of immunoglobulins, estimation of antibody titre Scand J Immunol 1973 56 Suppl 161 164 10.1111/j.1365-3083.1973.tb03798.x 4204108

[B38] Axen R Porath J Ernback S Chemical coupling of peptides and proteins to polyssacharides by means of cyanogen halides Nature 1967 56 1302 1304 10.1038/2141302a0 6056841

[B39] Okuyama MW Sousa NM Beckers JF BioLiège: University of Liège-RWTH Aachen University Characteristics of pregnancy-associated glycoprotein (PAG)-like proteins in red deer Proceedings of Biomedica European Life Sciences Summit: 19 June 2013; Aachen 2013 16

[B40] Wilker C Ball B Reimers T Sasser G Brunner M Alexander B Giaquinto M Use of pregnancy-specific protein-B and estrone sulfate for determination of pregnancy on Day 49 in Fallow deer (*Dama dama*) Theriogenology 1993 56 307 312 10.1016/0093-691X(93)90268-A 16727316

[B41] Willard ST Sasser RG Jaques JT White DR Neuendorff DA Randel RD Early pregnancy detection and the hormonal characterization of embryonic-fetal mortality in Fallow deer (*Dama dama*) Theriogenology 1998 56 861 869 10.1016/S0093-691X(98)00035-1 10732094

[B42] Willard ST Petty SJ Sasser RG White DR Randel RD Pregnancy detection and the effects of age, body weight, and previous reproductive performance on pregnancy status and weaning rates of farmed Fallow deer (*Dama dama*) J Anim Sci 1999 56 32 38 10064025 10.2527/1999.77132x

[B43] Szafranska B Panasiewicz G Majewska M Biodiversity of multiple pregnancy-associated glycoprotein (PAG) family: gene cloning and chorionic protein purification in domestic and wild eutherians (Placentalia) - a review Reprod Nutr Dev 2006 56 481 502 10.1051/rnd:2006034 17107639

[B44] Zhang WQ Zhang MH Phylogeny and evolution of Cervidae based on complete mitochondrial genomes Gen Mol Res 2012 56 628 635 10.4238/2012.March.14.6 22535398

[B45] Doré JJE Kattesh HG Godkin JD Isolation and identification of porcine embryonic basic protein as a fragment of pregnancy-associated glycoprotein-2 Int J Biochem Cell Biol 1996 56 1249 1255 10.1016/S1357-2725(96)00054-4 9022284

[B46] Mizejewski GJ The phylogeny of alpha-fetoprotein in vertebrates: survey of biochemical and physiological data Crit Rev Eukaryotic Gene Expres 1995 56 281 316 10.1615/CritRevEukarGeneExpr.v5.i3-4.40 8834228

[B47] Pedersen KO Fetuin, a new globulin isolated from serum Nature 1944 56 575

[B48] Spiro RG Studies on fetuin, a glycoprotein of fetal serum. I. Isolation, chemical composition, and physiochemical properties J Biol Chem 1960 56 2680 2689 16479679

[B49] Janzen RG Mably ER Tamaoki T Church RB Lorscheider FL Synthesis of alpha-fetoprotein by the pre-implantation and post-implantation bovine embryo J Reprod Fert 1982 56 105 110 10.1530/jrf.0.0650105 6176710

[B50] Denecke B Gräber S Schäfer C Heiss A Wöltje M Jahnen-Dechent W Tissue distribution and activity testing suggest a similar but not identical function of fetuin-B and fetuin-A Biochem J 2003 56 Pt 1 135 145 12943536 10.1042/BJ20030676 PMC1223762

[B51] Lai PC Huang LL Panrucker DE Church RB Lorscheider FL Distribution of bovine fetuin and albumin in plasma, allantoic and amniotic fluids during development J Reprod Fert 1981 56 53 60 10.1530/jrf.0.0630053 6168760

[B52] Dziegielewska KM Brown WM Gould CC Matthews N Sedgwick JE Saunders NR Fetuin: an acute phase protein in cattle J Comp Physiol B 1992 56 168 171 10.1007/BF00398343 1375608

[B53] Mizejewski GJ Alpha-fetoprotein structure and function: relevance to isoforms, epitopes, and conformational variants Exp Biol Med (Maywood) 2001 56 377 408 11393167 10.1177/153537020122600503

[B54] Peters TJ Serum albumin: recent progress in the understanding of its structure and biosynthesis Clin Chem 1977 56 5 12 318940

[B55] Roberts RM Xie S Mathialagan N Maternal recognition of pregnancy Biol Reprod 1996 56 294 302 10.1095/biolreprod54.2.294 8788179

[B56] Telugu BP Palmier MO van Doren SR Green JA An examination of the proteolytic activity for bovine pregnancy-associated glycoproteins 2 and 12 Biol Chem 2010 56 259 270 20030586 10.1515/BC.2010.016 PMC2969838

